# Cardiac magnetic resonance imaging for the detection of myocardial involvement in granulomatosis with polyangiitis

**DOI:** 10.1007/s10554-020-02066-2

**Published:** 2020-10-14

**Authors:** Alessandro Giollo, Raluca B. Dumitru, Peter P. Swoboda, Sven Plein, John P. Greenwood, Maya H. Buch, Jacqueline Andrews

**Affiliations:** 1 NIHR Leeds Biomedical Research Centre and Clinical Research Facility, Leeds Teaching Hospitals NHS Trust, Leeds, UK; 2 Leeds Institute of Rheumatic and Musculoskeletal Medicine, University of Leeds, Leeds, UK; 3 Leeds Institute of Cardiovascular and Metabolic Medicine, University of Leeds, Leeds, UK; 4 Rheumatology Section, Department of Medicine, University of Verona, Policlinico G.B. Rossi 10, 37134 Verona, Italy; 5 Centre for Musculoskeletal Research, University of Manchester, Manchester, UK; 6 NIHR Manchester Biomedical Research Centre, Manchester Teaching Hospitals NHS Trust, Manchester, UK

**Keywords:** Vasculitis, Granulomatosis with polyangiitis, Cardiovascular disease, CMR, ANCA, Myocardial fibrosis, LGE

## Abstract

The prevalence of undiagnosed cardiac involvement in granulomatosis with polyangiitis (GPA) is unknown. In this prospective study we investigated the utility of cardiovascular magnetic resonance (CMR) to identify myocardial abnormalities in GPA and their correlation with disease phenotype. Twenty-six patients with GPA and no cardiovascular disease or diabetes mellitus underwent contrast-enhanced CMR, including late gadolinium-enhancement (LGE), T1-mapping for native T1 and extra-cellular volume (ECV) quantification for assessment of myocardial fibrosis, cine imaging and tissue tagging for assessment of left ventricular (LV) function. Twenty-five healthy volunteers (HV) with comparable age, sex, BMI and arterial blood pressure served as controls. Patients with GPA had similar cardiovascular risk profile to HV. A focal, non-ischaemic LGE pattern of fibrosis was detected in 24% of patients and no controls (p = 0.010). Patients with myocardial LGE were less frequently PR3 ANCA (7% vs 93%, p = 0.007), and had involvement of the lower respiratory tract and skin. LGE scar mass was higher in patients presenting with renal involvement. Native T1 and ECV were higher in patients with GPA than HV; ECV was higher in those with relapsing disease, and native T1 was inversely associated with PR3 ANCA (β = − 0.664, p = 0.001). Peak systolic strain was slightly reduced in GPA compared to controls; LV ejection function was inversely correlated with disease duration (β = − 0.454, p = 0.026). Patients with GPA have significant myocardial abnormalities on CMR. ANCA, systemic involvement and disease severity were associated with myocardial fibrosis. CMR could be a useful tool for risk stratification of myocardial involvement in GPA.

**Electronic supplementary material:**

The online version of this article (10.1007/s10554-020-02066-2) contains supplementary material, which is available to authorized users.

## Introduction

Granulomatosis with polyangiitis (GPA), a systemic inflammatory disease, is an ANCA-associated small-vessel vasculitides (AAV) [1] with significant morbidity and mortality [2, 3]. Recent epidemiological studies have highlighted the increased frequency of cardiovascular (CV) events in patients with AAV and GPA [4–6], with resulting higher death rates from CV disease (CVD) [7, 8].

The increased morbidity and mortality due to CVD in patients with immune-mediated and inflammatory diseases (IMID) is thought primarily to be due to accelerated atherosclerosis [9]. However, primary myocardial involvement due to inflammation unrelated to typical atherosclerotic processes has been described in patients with systemic vasculitides [10–14], despite modern era immunosuppression [15].

Despite clear evidence of adverse CV outcomes in GPA, the underlying pathogenesis for cardiovascular involvement in GPA is poorly understood. Inflammatory myocardial involvement is probably underestimated as it is often not clinically looked for or detected until a CV event occurs. A retrospective echocardiography study revealed CV abnormalities related to GPA in up to 36% of patients [16]. Although the impact of subclinical CVD in GPA is not yet known, in the general population it has been associated with an increased risk of CV events in large population studies [17, 18].

Cardiovascular magnetic resonance (CMR) is a non-invasive imaging technique that is increasingly used to detect CVD in patients with IMID [19] with clear advantages over echocardiography. In particular, CMR is highly accurate and reproducible for the evaluation of left ventricular (LV) volumes, function and mass, and for advanced myocardial tissue characterization [20]. In contrast to echocardiography, CMR imaging with late gadolinium enhancement (LGE) has the ability to detect focal myocardial fibrosis [21]. Myocardial T1 mapping methods with calculation of extracellular volume fraction (ECV) can be useful in detecting oedema and early subclinical, diffuse myocardial fibrosis, with good correlation to histological findings of myocardial fibrosis in various clinical contexts [22, 23].

Although preliminary reports suggest that CMR is valuable in AAV [24, 25], a systematic assessment of GPA patients with CMR imaging has not been performed. The primary objective of this single-centre, observational, prospective study was to determine the prevalence and pattern of subclinical cardiac involvement in GPA as detected by CMR imaging; the secondary objective was to describe which GPA disease characteristics were associated with cardiac abnormalities detected by CMR.

## Methods

### Study design

This study was a single-centre, observational, cross-sectional feasibility study. Consecutive patients between 18 and 80 years attending the Vasculitis clinic at Leeds Teaching Hospitals NHS Trust (LTHT) were approached to enter the study. In addition to a comprehensive clinical evaluation at baseline, participants underwent contrast-enhanced CMR. All patients gave their written, informed consent to take part in the study with approval of the National Research Ethics Service (10/H1306/88).

### Study population

All patients invited to participate met the 2012 revised Chapel Hill Consensus nomenclature for vasculitis [26]. Selection criteria was age 18-80, complete remission with a Birmingham Vasculitis Activity Score (BVAS) < 0 following induction therapy and no prior diagnosis of CVD or diabetes mellitus to minimise the possibility of subclinical CVD occurring due to multimorbidity. Controls were identified from a previously recruited pool of 80 healthy volunteers (HV) with no diabetes mellitus (DM) and no known CVD with their imaging assessments recorded using the same CMR scanner as the vasculitis subjects. The controls were frequency-matched to GPA patients according to age, sex, BMI and mean arterial pressure (MAP). All participants (both vasculitis subjects and HV) were excluded if they had DM, uncontrolled arterial hypertension or a prior diagnosis of CVD including: heart failure (HF), coronary artery disease (CAD), cerebrovascular accident (CVA), valve disease, and peripheral arterial disease (PAD).

### Clinical data

Comprehensive demographic and clinical data were collected including the following CV risk factors: Hypertension was defined as a systolic blood pressure (SBP) of at least 140 mmHg and/or a diastolic blood pressure (DBP) of at least 90 mmHg, and/or pharmacologically treated blood pressure of unknown cause. Obesity was identified as a BMI ≥ 30 kg/m^2^. Dyslipidaemia was defined as levels of total serum cholesterol (TC) above 190 mg/dl and/or triglycerides (TG) above 150 mg/dl, or pharmacologically treated high lipid serum levels. To assess renal function, we considered the glomerular filtration rate (GFR) estimated with the chronic kidney disease-epidemiology (CKD-EPI) equation.

GPA assessment was recorded as disease phenotype (organ involvement, disease duration, ANCA status, number of relapses), past and current medications, disease activity assessed by BVAS, 3rd version [27] and damage assessed by the Vasculitis Damage Index (VDI) [28].

Disease relapse was defined as reappearance or worsening of vasculitis symptoms requiring an increase of the current treatment or introduction of additional immunosuppressive medication.

### Biochemical assessment

Blood samples were collected in close proximity to the day of CMR assessment. The following measurements were performed: full blood counts (FBC), creatinine (P-Cr), blood urea nitrogen (BUN), anti-neutrophil cytoplasm antibodies (ANCA), C-reactive protein (CRP), and erythrocyte sedimentation rate (ESR).

### CMR protocol and analysis

CMR studies were performed on a 3T Philips Achieva MR system. Trained CMR radiographers directed the scan, and image analysis was performed in accordance with recognised reporting standards [29] and using established and validated protocols [30]. The full CMR protocol included resting wall motion and LV function, tissue tagging, aortic distensibility, LGE, native and post contrast T1 mapping for ECV quantification. Native T1 and ECV were measured in areas without scar on LGE. Comprehensive and detailed descriptions of each analysis and their interpretation are available in the on-line supplementary file. LGE analysis was performed by two independent assessors (RBD, JG) and quantified using the Full Width Half Maximum (FWHM) technique. Aortic distensibility was defined as decreased if below the 5th centile of a reference population [31]. Normal ranges for ECV was defined as increased if above the upper limit of a reference range previously reported [32].

### Statistical analysis

As a feasibility study, formal power calculation for determining sample size was not indicated. Descriptive statistics are reported. Continuous data are represented by median [25th, 75th percentile]. Categorical data are described as absolute number (percentage). Between groups comparisons of continuous variables, including differences between GPA patients and heathy volunteers, were performed with the Mann–Whitney U test. Associations between dichotomous variables were assessed by Chi squared test or Fisher’s exact test where appropriate. The strength of the association between disease characteristics, CV risk factors and continuous CMR measures were assessed by the Spearman’s rho (r) coefficients. The statistical package IBM SPSS Statistics for Windows, Version 20.0 (Armonk, New York, USA) was used for the matching procedure and statistical analysis. Statistical significance was considered for a P value of 0.05 or less.

## Results

### Patient disposition

**Fig. 1:**
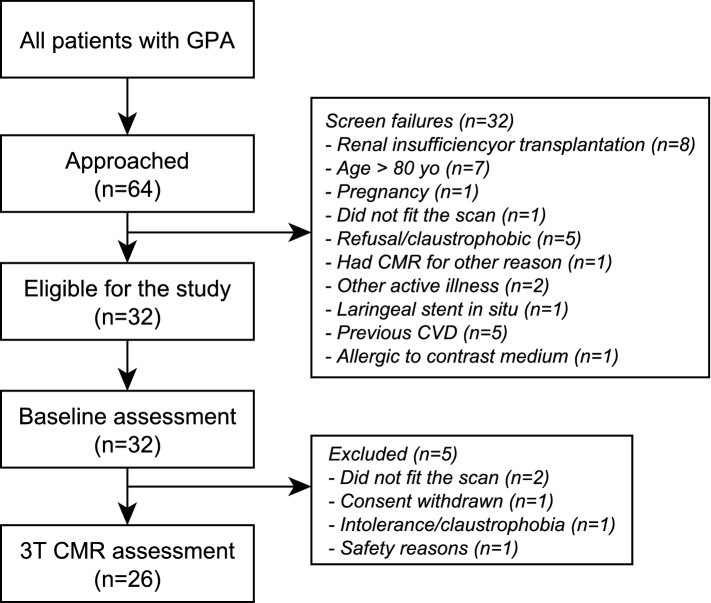
Flow chart of GPA patients participating in the study

Sixty-four patients were consecutively approached between October 2017 and April 2018 from which 32 patients consented to enter the study and 26 patients proceeded to CMR assessment (patient flow pathway is illustrated in Fig. 1). One patient did not receive gadolinium-based contrast due to their inability to tolerate the intravenous (IV) infusion.

### Baseline characteristics of study participants

The baseline patient characteristics are reported in Table 1. Median age of GPA patients was 58 years and all but one of the patients were white British. All patients had established disease as per study protocol, three-quarters were ANCA positive, and almost all received either CYC or RTX for induction of remission. ENT, pulmonary and renal involvement was frequent (over 60%), and the majority had suffered at least one disease relapse.

**Table 1:** Disease characteristics of patients with AAV

Disease characteristics	GPA patients (n = 26)
Disease duration (year)	8 (2, 12)
Biopsy obtained [n(%)]	20 (77)
ANCA-positive at diagnosis [n (%)]	20 (77)
IFI	22 (85)
ANCA PR3	15 (58)
ANCA MPO	6 (23)
Organ involvement [n (%)]	
Ear, nose, throat	22 (85)
Pulmonary	13 (50)
Renal	13 (50)
Musculoskeletal	12 (46)
Cutaneous	9 (35)
Ophthalmological	8 (31)
Neurological	7 (27)
Gastrointestinal	0 (0)
Induction treatment [n (%)]	
Cyclophosphamide	18 (69)
Rituximab	5 (19)
Other	3 (12)
Relapsing disease [n (%)]	17 (65)
Last relapse (months)	42 (10,91)
Current treatments [n (%)]	26 (100)
Methotrexate	5 (19)
Mycophenolate	3 (12)
Azathioprine	8 (31)
RTX last cycle (months)	28 (9, 50)
CYC last cycle (months)	15 (7, 77)
Prednisolone	11 (42)
Prednisolone dose (mg daily)	5 (5, 10)
Disease activity	
BVAS	2 (1, 4)
Remission (BVAS = 0) [n (%)]	6 (23)
VDI	1 (0, 1)

Median (25th, 75th percentile) values are presented unless stated otherwise

*ANCA* Anti-neutrophil cytoplasmic antibody, *BMI* body mass index, *BP* blood pressure, *BVAS* Birmingham Vasculitis Activity Score, *CV* cardiovascular disease, *CRP* C-reactive protein, *CYC* cyclophosphamide, *ESR* erythrocyte sedimentation rate, *RTX* rituximab, *VDI* vasculitis damage index

With regards to CV profile (Table 2), 19/26 (73%) patients had at least one (median 1 [0, 3]) traditional CV risk factor. Approximately half of the study population was taking CV medications. As per protocol, heathy volunteers did not differ significantly from patients with GPA when compared for age, BMI, BP and the male-to-female ratio.

**Table 2:** Cardiovascular risk factors of study population

Cardiovascular risk factors and medications	GPA patients (n = 26)	Heathy volunteers (n = 25)	P value
Age (years)	58 (45, 66)	61 (51, 66)	0.665
Female sex [n (%)]	12 (46)	7 (28)	0.180
Smoking status (ever)	5 (19)	0 (0)	0.021
BMI [kg/m^2^]	28.7 (24.3, 30.8)	27.0 (23.9, 28.9)	0.175
Obese [n (%)]	9 (35)	5 (20)	0.242
Hypertension [n (%)]	10 (39)	7 (28)	0.556
SBP [mmHg]	137 (121, 141)	122 (117, 142)	0.127
DBP [mmHg]	76 (67, 82)	72 (61, 79)	0.279
MAP [mmHg]	94 (86, 103)	90 (82, 96)	0.152
Dyslipidaemia [n (%)]	9 (35)	NA	NA

Median (25th, 75th percentile) values are presented unless stated otherwise

*BMI* body mass index, *BP* blood pressure, *CV* cardiovascular disease, *CRP* C-reactive protein, *ESR* erythrocyte sedimentation rate, *LV* left ventricular, *MAP* mean arterial pressure, *MCV* mean corpuscular volume, *NA* data not available

### Comparison of CMR measures between GPA subjects and heathy volunteers

**Fig. 2:**
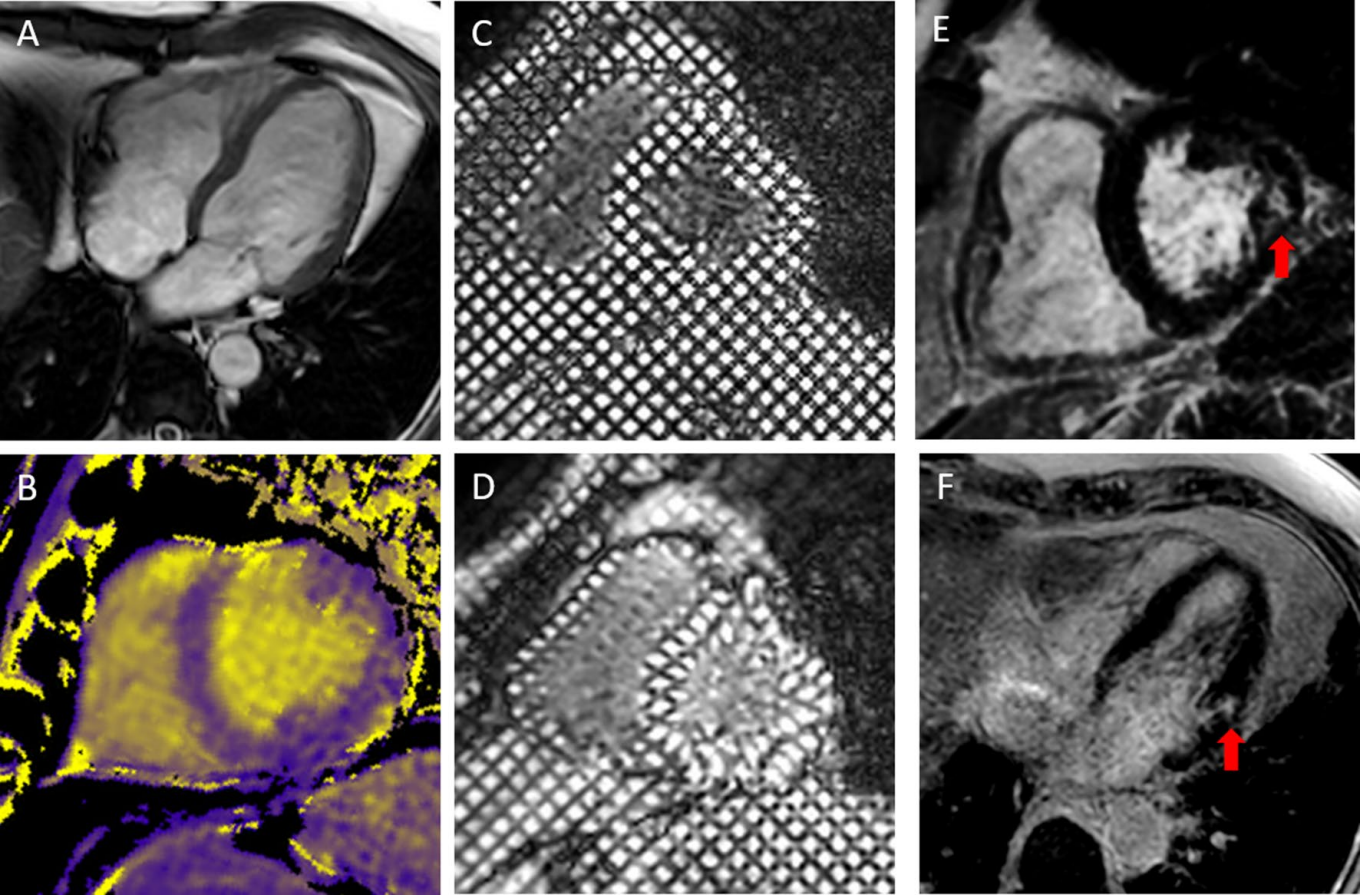
CMR imaging from a single patient with GPA: SSFP cine imaging (**a**), Native T1 map with colour scale from 1000 ms (purple) to 2000 ms (yellow) (**b**), grid tags at end diastole (**c**) and end systole (**d**) and late gadolinium enhancement imaging in short (**e**) and long axis (**f**) with focal fibrosis shown by red arrow

Compared to heathy volunteers, GPA patients had smaller chamber volumes and decreased LV mass. Native T1 and ECV were significantly higher in GPA than controls (6/25 patients had increased ECV, no HV had increased ECV). LGE was detected in 8/25 (32%) GPA patients and in no controls. No GPA patient had LGE in a pattern of enhancement suggesting ischaemic-mediated myocardial damage. A focal pattern of fibrosis was seen in all GPA patients with LGE. LGE most commonly involved the basal LV and lateral segments and was located in the midwall to subepicardial layer in 6/8 (75%) patients (Table 3; Fig. 2). Two patients had evidence of scarring uniquely in the inferior wall and right ventricle insertion point (RVIP). However, the pathogenesis and clinical relevance of LGE at RVIP remains poorly understood, and is generally believed to be a nonspecific finding in CMR studies, although a few reports suggest that RVIP-LGE may be related to pulmonary hypertension [33–36]. Hence, when subsequently we analysed correlations and associations of clinical variables with LGE, we chose to exclude the two patients with isolated RVIP-LGE, as we believed it had a low likelihood to represent a distinct pattern of myocardial fibrosis in our patients with GPA. There was no difference in LV EF between patients with GPA and heathy volunteers (Table 3), and values for strain were within normal range both in patients and healthy volunteers.

**Table 3:** Comparison of CMR characteristics between GPA patients and heathy volunteers

CMR measures	GPA patients (n = 26)	Heathy volunteers (n = 25)	P value
LV volumes and mass			
LV EDV index (mL/m^2^)	69 (61, 84)	79 (70, 89)	0.105
LV ESV index (mL/m^2^)	28 (23, 37)	32 (28, 39)	0.122
LV mass index (g/m^2^)	37 (42, 47)	50 (42, 56)	0.014
LV mass/LVEDV, g/mL	0.59 (0.48, 0.66)	0.59 (0.54, 0.66)	0.486
LV function			
LV EF (%)	59 (56, 63)	58 (53, 62)	0.351
LV peak systolic strain			
Base	-0.19 (-0.21, -0.16)	-0.21 (-0.22, -0.19)	0.017
Mid	-0.21 (-0.23, -0.20)	-0.22 (-0.23, -0.20)	0.298
Apex	-0.21 (-0.23, -0.16)	-0.23 (-0.25, -0.20)	0.101
Torsion (°)	12.3 (9.2, 14.5)	13.8 (11.6, 16.0)	0.086
Peak twist^a^	14.1 (9.0, 15.7)	14.1 (11.7, 17.7)	0.222
Myocardial tissue characterization			
Native T1 (ms)	1225 (1199, 1255)	1203 (1185, 1233)	0.006
Extracellular volume (%)^b^	25.0 (23.3, 27.2)	23.6 (20.5, 25.1)	0.009
Increased extracellular volume [n (%)]	6/25 (24)	0 (0)	0.022
LGE [n (%)]^b^	8/25 (32)	0/25 (0)	0.010
LGE distribution			
Subepicardial	4/8 (50)	0/25 (0)	NA
Midwall	2/8 (25)	0/25 (0)	NA
RVIP	2/8 (25)	0/25 (0)	NA
LGE segments			
Basal antero-lateral	4/8 (50)	0/25 (0)	NA
Basal infero-lateral	3/8 (38)	0/25 (0)	NA
Basal antero-septal	1/8 (13)	0/25 (0)	NA
LGE scar tissue (g)	1.25 (0.81, 2.63)	0 (0, 0)	NA
Aortic distensibility			
Descending aorta (10^−3^ mmHg^−1^)	2.95 (2.29, 3.64)	NA	NA
Abnormal stiffness [n (%)]	19/26 (73)	NA	NA

Median (25th, 75th percentile) values are presented unless stated otherwise

Left and right ventricle volumes are indexed by body surface area

*CMR* cardiovascular magnetic resonance, *ECV* extracellular-volume fraction, *EDV* end-diastolic volume, *ESV* end-systolic volume, *LGE* late gadolinium enhancement, *LV* left ventricular, *LV EF* left ventricular ejection fraction, *NA* data not available, *RVIP* right ventricle insertion point

^a^Data available in 24/26 GPA patients

^b^Data available in 25/26 GPA patients

### Relationship between traditional CV risk factors and CMR measures

In the whole study population, diastolic BP was associated with LVEDV index independent of age and sex (β = − 0.29, p = 0.042). In patients with GPA, the descending aorta distensibility was decreased compared to reference values in 19/26 (73%; 100% of females and 50% of males) and correlated inversely with age (r = − 0.532, p = 0.005), LGE scar mass (r = − 0.810, p = 0.015), MAP (r = − 0.519, p = 0.007) and SBP (r = − 0.650, p < 0.001). We did not find significant associations between other CMR measures and CV risk factors.

### Associations of CMR measures with disease characteristics of GPA

LVESV index was associated with PR3 ANCA status (23 ml/m^2^ [21, 34] in PR3 ANCA negative *vs* 31 ml/m^2^ (26, 38) in PR3 ANCA positive patients, p = 0.032) and correlated with disease duration (r = 0.420, p = 0.033). LVEF was positively correlated to disease duration as well (r = − 0.429, p = 0.029). Native T1 was associated with PR3 ANCA status (1268 ms [1248, 1337]) in PR3 ANCA negative *vs* 1212 ms [1196, 1236] in PR3 ANCA positive patients, p < 0.001). ECV was significantly higher in GPA patients with relapsing disease (25.6% [24.2, 29.8] vs 24.1% [22.1, 25.8], p = 0.037). All results via univariate analysis.

### Characteristics of GPA patients with and without LGE

Patients with myocardial LGE (LGE+) were more frequently PR3 ANCA negative (93% vs 7%, p = 0.007), and more likely to have involvement of the lower respiratory tract (75% vs 25%, p = 0.097) and skin (63% vs 38%, p = 0.087). No significant differences were noted in previous or current immune-suppressive regimes, although there was a trend to less frequent use of RTX in LGE + patients (25% vs 75%, p = 0.081). Native T1 values were higher in LGE + patients: 1259 ms [1234, 1284] *vs* 1220 ms [1197, 1270], p = 0.023, and ECV was only modestly increased in this group. Patients with GPA and LGE + had a significantly higher LV mass/LVEDV ratio: 0.64 [0.60, 0.67] *vs* 0.52 [0.47, 0.63], p = 0.043. The LGE scar mass was associated with renal involvement: 0.82 g [0.80, 1.09] *vs* 2.98 g [1.58, 4.51], p = 0.036.

## Discussion

To our knowledge, this is the first prospective, multi-parametric CMR imaging study in GPA patients with no previous diagnosis of CVD or diabetes mellitus. As a feasibility study, it was not powered to detect significant associations between CMR outcomes and GPA disease characteristics, however we believe our results provide new insights into the prevalence and distribution of primary subclinical CVD in GPA and align with our findings using similar CMR protocols in other IMID cohorts [38, 39].

Few studies have investigated cardiac involvement in GPA using CMR imaging. One retrospective, non-controlled study with 1.5T CMR by the French Vasculitis Study Group [24] recruited over an 8-year period and did not comprehensively exclude concomitant CVD. Other investigators [25] performed a 1.5T CMR imaging with T1 and T2 mapping study in consecutive AAV patients and age and sex matched healthy volunteers. The authors reported a pooled analysis of AAV which included several patients with eosinophilic GPA (EGPA), a subset of AAV characterised by a distinct type of myocardial involvement. Current management guidelines of GPA do not recommend CV imaging at disease onset nor during follow up, although CVD is a major determinant of mortality in AAV [40], however the specific cardiac abnormalities associated with GPA have not been defined.

The primary finding of this feasibility study is the detection of myocardial fibrosis in approximately one-third of GPA patients, in line with previous reports [24, 25, 41]. LGE corresponds to areas of myocardial fibrosis as shown by comparison with histopathology [42], and its presence has prognostic value in ischaemic and non-ischaemic cardiomyopathies [43, 44]. The myocardial LGE can show different patterns according to diverse pathology; trans-mural LGE is typical of myocardial infarction whilst non-ischemic conditions associate with focal enhancement. Interestingly, the LGE seen in our GPA patients was focal and distributed subepicardially or at the mid-wall in most cases, in keeping with a non-ischaemic cause. Furthermore, this fibrotic tissue was frequently found in the lateral segments.

The presence of LGE was not associated with traditional CV risk factors, and we minimised the likelihood of underlying undiagnosed CAD by excluding GPA patients at highest risk (i.e. those with previous CV events and/or with DM). LGE was associated with PR3 ANCA negativity and also renal involvement, suggesting a potential relationship between myocardial fibrosis in GPA and specific disease characteristics. This finding is of clinical interest as the presence or absence of LGE on CMR could, in the future, help with CVD risk stratification in patients with GPA [19]. Whether myocardial LGE is associated with increased risk of relapse or disease severity needs to be addressed with further research.

Native T1 and ECV are indicative of a diffuse intramyocardial fibrosis/inflammation, including in the absence of infarction [45]. Native T1 and ECV values were slightly elevated in patients with GPA when compared to healthy volunteers, in keeping with previous reports [25]. We only included patients with established GPA, suggesting that a degree of cardiac inflammation is present throughout the GPA disease continuum, and, in our cohort, an association between native T1 and vasculitis-related damage (VDI) and inflammation (CRP).

Assessment of patients with GPA using CMR may also reveal LV remodelling, with characteristics similar to that seen in our previously reported IMID cohorts [37, 38]. LV volumes were decreased in GPA patients compared to healthy volunteers, similar findings have been reported with echocardiography in RA patients, often in association with a concentric LV geometry and subclinical LV dysfunction [46–48]. Interestingly, LV mass in GPA patients was also significantly lower than controls and below published normal ranges [45]; which has been previously reported in patients with AAV [25] and also systemic sclerosis [39] and RA [38], implying the presence of inflammatory myocardial involvement across the IMID spectrum.

Cardiac abnormalities are common in GPA patients and the likelihood of cardiac damage may be highest in the early stages of the disease [49, 50]. In this study we report primary, non-ischaemic myocardial abnormalities in asymptomatic GPA patients with well controlled disease, suggesting that subclinical cardiac pathology cay occur in GPA patients despite the absence of cardiac signs or symptoms [8]. The clinical implications of this remain to be determined. Additionally, in our study, a longer disease duration was associated with several measures of LV systolic dysfunction, underlining the importance of aiming for disease remission rather than low disease activity in GPA. In the future, disease activity indexes could be used to aid the prediction of future CV events and CVD-related mortality in patients with GPA [51].

### Clinical implications and novelties

In this study, we prospectively demonstrated the feasibility and usefulness of CMR in GPA. Our data show that patients with GPA have both subclinical inflammation and fibrosis detected by CMR mapping techniques, despite being free of overt cardiac disease and vasculitic symptoms. ECV was associated with relapsing disease, suggesting that a clinically non-resolving vasculitis may cause active myocardial inflammation. Moreover, we demonstrated that myocardial fibrosis was present even when ANCA were not detectable in patients with skin and pulmonary involvement from vasculitis. Hence, our data could help to define those patients clinically at a higher risk of cardiac involvement. T1 mapping, ECV and LGE should all be assessed for the characterization of myocardial involvement in GPA, as they provide complementary information about diffuse myocardial involvement compared to one technique alone. Whether such alterations are reversible or can progress to additional damage should be addressed in further research.

### Study strengths and limitations

Major strengths include: first, the pre-defined protocol with 3.0T CMR imaging, which informed rheumatologists to perform T1 mapping, LGE and ECV to detect myocardial involvement in GPA; second, the inclusion of controls also matched for arterial BP, which allowed to exclude hypertensive cardiac alterations; third, the multidisciplinary nature of our research group, which includes clinician-researchers experienced in both vasculitis and cardiac imaging; and last, we fully described the vasculitis status and characteristics and their associations with CMR abnormalities. Finally, our study included mostly patients with low disease activity (median BVAS = 2), actually showing that subclinical myocardial involvement can be detected even in GPA patients without active significant organ involvement.

The most important limitation of our study is a small size, though it is comparable to previous research groups reporting on the same topic. We advocate multi-centric studies to increment the study population and to validate our results. Another limitation is that we could not entirely exclude CAD, yet we excluded a priori participants with overt CVD, diabetes or uncontrolled hypertension. Finally, a validation procedure, such as endomyocardial biopsy, was not performed. Endomyocardial biopsy was not offered to patients as they all had preserved LV-EF and were all asymptomatic for cardiac symptoms. However, endomyocardial biopsy has several limitations, including low sensibility, lowering its diagnostic benefit.

## Conclusion

This study describes CMR imaging findings in patients with established stable GPA. Myocardial fibrosis and abnormal LV geometry were more common in GPA patients than in matched healthy controls, and correlate with certain disease characteristics. Although the prognostic implications of our results are yet to be fully understood, our group has reported on the frequency and severity of cardiac abnormalities in a population of GPA patients which will inform future studies aimed at evaluating the management of cardiac involvement in patients with GPA.

## Electronic supplementary material

Below is the link to the electronic supplementary material.

Supplementary material 1 (DOCX 35 kb)
