# Supplementary material for: Cardiac magnetic resonance imaging for the detection of myocardial involvement in granulomatosis with polyangiitis
Source: Int J Cardiovasc Imaging. 2020 Oct 14;37(3):1053–62. doi: 10.1007/s10554-020-02066-2 (PMC7969556; doi:10.1007/s10554-020-02066-2)
Supplement: Supplementary file 1 — Supplementary material 1 (DOCX 35 kb) [file 10554_2020_2066_MOESM1_ESM.docx]

## **CMR protocol**

The scans were performed on a 3T Philips Achieva MR system using a 32-channel receiver coil. The CMR protocol has been previously described [1, 2] and included:

Cine imaging

Cine imaging were acquired in the vertical long axis, short axis and horizontal long axis using a balanced steady state free precession [bSSFP] pulse sequence. Multi-slice, multi-phase cine image stack was acquired in the short axis to cover the entire left and right ventricle (10-12 contiguous slices, 30 cardiac phases, spatial resolution 2.0 × 1.63 × 8 mm^3^, TR 2.6 ms, TE 1.3 ms).

Tissue tagging

Tissue tagging imaging was generated using the ‘3-of-5’ approach [5] using spatial modulation of magnetization (SPAMM) pulse sequence (spatial resolution 1.51×1.57×10 mm,^3^ tag separation 7 mm, ≥18 phases, typical TR/TE 5.8/3.5 ms).

Aortic distensibility

For aortic distensibility, a multi-phase SSFP cine imaging, transverse to the ascending and descending aorta, at the level of the of the pulmonary artery bifurcation was used (retrospective gating, slice thickness 10 mm, 50 phases, acquired spatial resolution 1.2 × 1.2 mm, repetition time 2.6 ms, echo time 1.3 ms). Brachial blood pressure was measured prior to image acquisition

T1 mapping

T1 mapping was performed using an ECG-triggered modified Look-Locker inversion (MOLLI) sequence. The ‘3-of 5’ approach was used [3, 4]. Post-contrast T1 maps were acquired 15 minutes after contrast administration (11 images, 3-3-5 acquisition, 3x R-R interval recovery epochs, voxel size 1.7 x 2.14 x 10 mm³ trigger delay at end-diastole, flip angle 35^o^, FOV of 320 – 420 mm).

Late gadolinium enhancement (LGE)

LGE imaging was performed in 10-12 short slices, 10-15 minutes following gadolinium administration (0.1mmol/kg Magnevist, Bayer), using an inversion recovery-prepared T1-weighted gradient echo (TE 2.0 ms, TR 3.5 ms, spatial resolution 1.54 × 1.76 × 10 mm^3^). A Look-Locker sequence was used to find the optimal inversion time to null the myocardium. Cross-cuts and phase swap were generated to confirm the presence of LGE.

.

## **CMR analysis**

CMR analysis was performed using dedicated computer software (cvi42, Circle Cardiovascular Imaging Inc., Calgary, Canada and InTag, CREATIS lab, Lyon, France). Left ventricle epicardial and endocardial borders were manually contoured on the LV short axis SSFP cine stack to determine LV volumes and function (papillary muscles and trabeculation excluded).

Tissue tagging was analysed using dedicated software (InTag, CREATIS lab, Lyon, France). The endocardial and epicardial contours were delineated for each slice and propagated throughout the cardiac phases using a semi-automated method. LV circumferential strain was measured for all three short axis slices. Basal and apical radius were determined from cine images in diastole. The following formula was applied to measure torsion, where LV peak twist is the difference between the basal and apical rotation.

$$Torsion=\frac{Peak twist \times(Apical Radius+Basal Radius)}{2\times Apex to Base length}$$

To determine aortic distensibility, cross-sectional measurements at the level of the descending aorta were made by manual planimetry of the endovascular-blood pool interface, at maximal and minimal distension. The following equation was used to calculate aortic distensibility (mmHg^−1^)[6].

$$Aortic distensibility=\frac{\Delta Aortic area}{Pulse pressure\times minimum aortic area}$$

Native and post-contrast myocardial T1 were calculated at the mid LV slice. Endocardial and epicardial contours were first delineated. The region of interest (ROI) was drawn in the mid interventricular septum[7], taking care to avoid partial-volume effects from the neighbouring tissue and areas that were hyperintense on LGE imaging. Extracellular volume (ECV) was calculated using the following formula, where R1 is 1/T1 [8].

$ECV =\left( 1-hematocrit \right)\times\frac{R1 myo post-R1 myo pre}{R1 blood post-R1 blood pre}$

LGE was assessed by two experienced CMR operators and reported according to the 16 segment American Heart Association (AHA) model [9]. Quantitative LGE assessment was performed using the full-width at half-maximum (FWHM) semi-automated method [10]. Epicardial and endocardial contours were delineated manually, and a ROI was drawn around the hyperintense myocardium and used to define maximal signal for the FWHM threshold. Care was taken to avoid partial volume effects from the neighbouring tissue and blood pool.

References

1. Bissell LA, Dumitru RB, Erhayiem B, Abignano G, Fent G, Kidambi A, et al. Incidental significant arrhythmia in scleroderma associates with cardiac magnetic resonance measure of fibrosis and hs-TnI and NT-proBNP. Rheumatology (Oxford). 2019 Jan 28.

2. Erhayiem B, Pavitt S, Baxter P, Andrews J, Greenwood JP, Buch MH, et al. Coronary Artery Disease Evaluation in Rheumatoid Arthritis (CADERA): study protocol for a randomized controlled trial. Trials. 2014 Nov 8; 15:436.

3. Messroghli DR, Greiser A, Frohlich M, Dietz R, Schulz-Menger J. Optimization and validation of a fully-integrated pulse sequence for modified look-locker inversion-recovery (MOLLI) T1 mapping of the heart. Journal of Magnetic Resonance Imaging. 2007 Oct; 26(4):1081-1086.

4. Messroghli DR, Radjenovic A, Kozerke S, Higgins DM, Sivananthan MU, Ridgway JP. Modified Look-Locker inversion recovery (MOLLI) for high-resolution T-1 mapping of the heart. Magnet Reson Med. 2004 Jul; 52(1):141-146.

5. Messroghli DR, Bainbridge GJ, Alfakih K, Jones TR, Plein S, Ridgway JP, et al. Assessment of regional left ventricular function: accuracy and reproducibility of positioning standard short-axis sections in cardiac MR imaging. Radiology. 2005 Apr; 235(1):229-236.

6. Oliver JJ, Webb DJ. Noninvasive assessment of arterial stiffness and risk of atherosclerotic events. Arterioscl Throm Vas. 2003 Apr; 23(4):554-566.

7. Rogers T, Dabir D, Mahmoud I, Voigt T, Schaeffter T, Nagel E, et al. Standardization of T1 measurements with MOLLI in differentiation between health and disease - the ConSept study. Journal of Cardiovascular Magnetic Resonance. 2013 Sep 11; 15(1):78.

8. Miller CA, Naish JH, Bishop P, Coutts G, Clark D, Zhao S, et al. Comprehensive validation of cardiovascular magnetic resonance techniques for the assessment of myocardial extracellular volume. Circulation Cardiovascular imaging. 2013 May 1; 6(3):373-383.

9. Cerqueira MD, Weissman NJ, Dilsizian V, Jacobs AK, Kaul S, Laskey WK, et al. Standardized myocardial segmentation and nomenclature for tomographic imaging of the heart. A statement for healthcare professionals from the Cardiac Imaging Committee of the Council on Clinical Cardiology of the American Heart Association. Circulation. 2002 Jan 29; 105(4):539-542.

10. Flett AS, Hasleton J, Cook C, Hausenloy D, Quarta G, Ariti C, et al. Evaluation of techniques for the quantification of myocardial scar of differing etiology using cardiac magnetic resonance. JACC Cardiovasc Imaging. 2011 Feb; 4(2):150-156.
